# Levels of evidence for human system risk evaluation

**DOI:** 10.1038/s41526-024-00372-w

**Published:** 2024-03-20

**Authors:** Jessica Ward, Robert J. Reynolds, Erin Connell, Wilma Anton, Avalon Kabeel, Jacqueline M. Charvat, Nicholas Nartey, Kristina Marotta, Ahmed Abukmail, Dan M. Buckland, Mary Van Baalen, Erik Antonsen

**Affiliations:** 1 Department of Emergency Medicine, Beth Israel Deaconess Medical Center, Boston, MA USA; 2 KBR, Houston, TX USA; 3 Leidos Innovations, Houston, TX USA; 4 JES Tech, Houston, TX USA; 5 NASA Pathways Intern, NASA Johnson Space Center, Houston, TX USA; 6 University of Houston-Clear Lake, Houston, TX USA; 7 Duke University, Durham, NC USA; 8 NASA Johnson Space Center, Houston, TX USA; 9 Department of Emergency Medicine, Center for Space Medicine, Baylor College of Medicine, Houston, TX USA

**Keywords:** Medical research, Aerospace engineering, Risk factors, Research data, Research management

## Abstract

NASA uses a continuous risk management process to seek out new knowledge of spaceflight-induced risk to human health and performance. The evidence base that informs the risk assessments in this domain is constantly changing as more information is gleaned from a continuous human presence in space and from ongoing research. However, the limitations of this evidence are difficult to characterize because fewer than 700 humans have ever flown in space, and information comes from a variety of sources that span disciplines, including engineering, medicine, food and nutrition, and many other life sciences. The Human System Risk Board (HSRB) at NASA is responsible for assessing risk to astronauts and communicating this risk to agency decision-makers. A critical part of that communication is conveying the uncertainty regarding the understanding of the changes that spaceflight induces in human processes and the complex interactions between humans and the spacecraft. Although the strength of evidence grades is common in the academic literature, these scores are often not useful for the problems of human spaceflight. The HSRB continues to update the processes used to report the levels of evidence. This paper describes recent updates to the methods used to assign the level of evidence scores to the official risk postures and to the causal diagrams used by the HSRB.

## Introduction

The enterprise of human spaceflight carries significant risk. The tragedies of the Challenger and the Columbia Space Shuttles highlight one aspect of that risk—the risk of vehicle failure. Spaceflight vehicles are extremely complicated machines, and early in the space program, potential vehicle failure was the greatest risk to humans flying in space^1^: however, astronauts also confront many other types of risks that the National Aeronautics and Space Administration (NASA) and commercial space companies must consider. The human body is also immensely complex, and the changes that occur to it during spaceflight, coupled with the complex interactions of humans with the spacecraft, introduce significant challenges to successful performance during a mission. The health risks induced by short-duration spaceflights do not contribute significantly to overall mission risk: the medical risk component was only a small fraction of the total risk for Space Shuttle astronauts^2^. Here, the term short-duration refers to missions <30 days in duration. However, as NASA and commercial companies look to return to the Moon and travel on to Mars, the risks to human health and performance become more pronounced^3–5^. Assessing the many potential spaceflight-induced risks to different human body systems (henceforth referred to as human systems risks) is a significant challenge. The Human System Risk Board (HSRB) at NASA is tasked with assembling a disparate set of experts to identify, assess, track, and report on the human system risks associated with spaceflight. The JSC 66705 Human System Risk Management Plan provides the background of this topic^6^.

At the time of this writing, there are 30 crew health and performance items (29 risks and 1 concern) managed by the HSRB in its risk portfolio on behalf of NASA^7,8^. The risks are derived from five spaceflight hazards: altered gravity, space radiation, isolation and confinement, hostile closed environment, and distance from Earth. These hazards are, to varying degrees, omnipresent for all spaceflight missions.

The HSRB has a responsibility to determine the reliability and level of evidence (LoE) that supports its assertions regarding risk posture. Risk posture refers to the likelihood (L) and consequence (C) of an undesired outcome. The product of L × C is the expectation of an outcome. The LoE, on the other hand, is an expression of certainty of belief in the hypothesized causal system that explains that risk. Insufficient predictive capability can be due to two sources of uncertainty. The first is the uncertainty inherent in a well-defined probabilistic process. The second is the uncertainty of our belief in how well-defined the probabilistic process is; this is due to insufficient knowledge^9^. The former is reflected in statistical measures of variation, while the latter is reflected in LoE. This is true for both the global assessment of risk posture for a specific risk as well as the causal mechanisms involved in directed acyclic graphs (DAGs). The DAG is a model of the causal mechanisms that lead from hazards to outcomes and is discussed more below. The HSRB processes for risk management and the description and use of DAGs are the topic of other papers in this collection^10^.

LoE is at the core of the relationship between risk management, knowledge management, and decision-making by NASA and helps to appropriately communicate epistemic uncertainty of spaceflight-induced risk for humans to program managers and engineers^11–13^. When making decisions about risk posture, the HSRB must ask the questions: How well do we understand the risks involved? Are we guessing or estimating? Are we fairly certain or absolutely confident? An understanding of the LoE that supports this awareness helps decision-making and is essential when humans reach the limits of their performance during the first human missions to Mars^6,11,14^.

In their prior approach to assessing LoE, the HSRB used the Romero and Francisco matrix to delineate four categories of evidence: cellular, animal, terrestrial, and spaceflight^15^. These categories were assigned a LoE scale that was primarily derived for epidemiologically structured evidence, and where the highest ranking category of LoE was *causation*, followed by *association*, *incidence*, *prevalence*, *case series*, and *case study*. However, during the years that this approach was used, several shortcomings were identified. First, the assignments to different LoEs bypassed an initial step that should be performed by a subject matter expert (SME) who critically evaluates the publications and data sources for limitations and applicability (this is the concept of Quality of Evidence (QoE) that will be discussed further in the next section). Occasionally, evidence sources that used inadequate study approaches or did not discuss the limitations of generalizing animal outcomes to human concerns were included in the evidence base without critical discussion of these limitations. This eventually led to the claim that the LoE was determined from all published data in the domain without a critical analysis of those works. In a field where evidence is often sparsely available and the generalizability of results is limited, critical analysis of data and publications as they relate to claims of risk posture and causality is essential to communicating a meaningful LoE. Second, in the prior LoE approach, no guidance was provided on what qualified as causal as opposed to association. Pearl and D. MacKenzie, in their work on counterfactual analysis, demonstrate that different approaches are required to establish causality^16^. Therefore, a formal risk assessment for human spaceflight should be based on an understanding of the mechanism and causal factors, and it is our intention to lay the groundwork for using those approaches here. Third, although the prior categories for sources of evidence differentiated between terrestrial and spaceflight human-derived evidence, they did not make this distinction for cellular and animal evidence. This caused confusion and failed to differentiate between the animal or cellular information gleaned from spaceflight and the information generated from experiments conducted in ground analogs of spaceflight. These issues often clouded how much weight was given to various evidence, so a new approach was derived to assign LoE for spaceflight risk assessment. This new approach is described below.

## Systematically evaluating data and evidence using the new approach

### Overview

Strength of evidence grading systems abound in the literature. These LoE scales help identify gaps in knowledge, especially gaps in terrestrial clinical research and evidence, where physicians rely on the clinical evidence base to help inform their decisions about guidelines or patient care. Human spaceflight, however, differs from terrestrial situations: a paucity of spaceflight data and subjects exist; double-blind randomized controlled trials are exceedingly rare in spaceflight due to challenges of using small sample sizes (making the data less accurate and less reliable); and complex logistical issues arise when testing humans who are concurrently carrying out operational responsibilities while in space^17^. Additionally, on Earth, except in rare circumstances, a baseline set of assumptions can be made about the environment affecting the patient experience. Unique spaceflight hazards induce different environmental challenges during spaceflight, which can decondition humans^3,8^. The spaceflight environment is a synergy of multiple environmental exposures that decondition and stress astronauts in ways not necessarily observed in analogs. Most of the currently available evidence of spaceflight risk is from missions lasting 6 months or less in low earth orbit. Evidence gathered from these missions may not be applicable to the long-duration lunar and planetary missions currently being planned. Additionally, because NASA is continuously developing vehicles, habitats, spacesuits, and systems that will impact the risk posture years prior to a given mission, the HSRB cannot wait for the evidence base to develop before providing risk assessments. The HSRB must evaluate all available evidence to the best of its ability while providing straightforward and meaningful methods of communicating the uncertainty in the knowledge base as it applies to risk management. This means that reviewing evidence and assigning scores such as QoE and LoE are not meant to be exhaustive but designed to walk the fine line between good enough for reasonable decision-making while respecting the resources and limitations that the evaluators must work within.

To address the unique challenges of assessing spaceflight risk, the HSRB has included two terms in their new approach: QoE and LoE, which indicate the level of certainty of understanding of the risk and the level of expectations to mitigate that risk. QoE is defined as “the extent to which all aspects of a study’s design and conduct can be shown to protect against systematic bias, nonsystematic bias, and inferential error”^18,19^. QoE applies most directly to an evaluation of published literature. When evaluating the evidence supporting HSRB assertions, SMEs are expected to evaluate multiple sources of information, including publications, systematic reviews, NASA evidence books, and unpublished internal agency data. This aggregation of information forms an evidence base that is relevant to the human system risk of interest. The QoE is then considered when determining the LoE, which is the strength of this aggregate evidence base and can be described as an assignment that “incorporates judgments of study quality and includes how confident one is that a finding is true”^18^. The “finding”, in this case, is the assertion made to the HSRB regarding the strength of the understanding of the risk.

The LoE scores support the high-level evaluation of NASA’s risk posture for each risk. The risk is communicated as either red (high), yellow (medium), or green (low) to align with other NASA risk approaches. Colors are determined using a risk matrix that can be found in the Human System Risk Management Plan document^6^. The HSRB uses the LoE to express uncertainty in the structure of DAGs for each risk^20,21^. These knowledge graphs start from at least one of the five spaceflight hazards (i.e. radiation, isolation and confinement, distance from Earth, altered gravity, hostile closed environment) that create each risk, then display the prominent contributing factors (e.g. physiological aspects, countermeasures) showing how they connect with each other and can lead to the mission level outcomes (e.g. loss of mission, long term health outcome). These relationships then form the basis of the high-level risk postures described elsewhere^6^. A standardized set of risk DAGs allows the HSRB to identify common touchpoints across risks that can identify potential synergistic relationships. This work proceeds in partnership with current efforts by the Human Research Program that seek to provide improved evidence on synergistic relationships among contributing factors to risk^22,23^. As evidence improves, the DAGs have the potential to be validated or disproven on the basis of that evidence^24^. A complete discussion of these DAGs is beyond the scope of this paper, more in-depth description can be found in the guidance documentation used by the HSRB^20^.

Many examples of the strength of evidence approaches exist, and they are specific to certain types of evidence, such as clinical or epidemiologic evidence. For example, in clinical evidence in terrestrial medicine, the Agency for Healthcare Research and Quality (AHRQ) has implemented a strength of evidence grading system that depends on five domains, including study limitations, directness, consistency, precision, and reporting bias^19,25^. This grading system is used to assess literature that has already been published. This system is appropriate for clinical studies and is relevant to human health in space. However, evidence issues faced in spaceflight are compounded by a variety of factors that can be different than terrestrial medicine including data challenges and unique spaceflight hazards. To assess QoE and LoE for a particular Human System Risk, we must discuss the sources of evidence and applications of these terms used by the HSRB.

### Sources of evidence

Sources of evidence relevant to human spaceflight risk must include information about both the human and the environment the human experiences. This data comes from a variety of sources, including clinical data derived from astronauts during flight, data derived during studies of exposures to environments or conditions analogous to spaceflight, and animal, molecular, cellular, and genetic data. Importantly, this also includes engineering data regarding the environment that is experienced during spaceflight, the trade space decisions that limit what systems and capabilities are present, and the likelihood of vehicle or system failures.

**Fig. 1:**
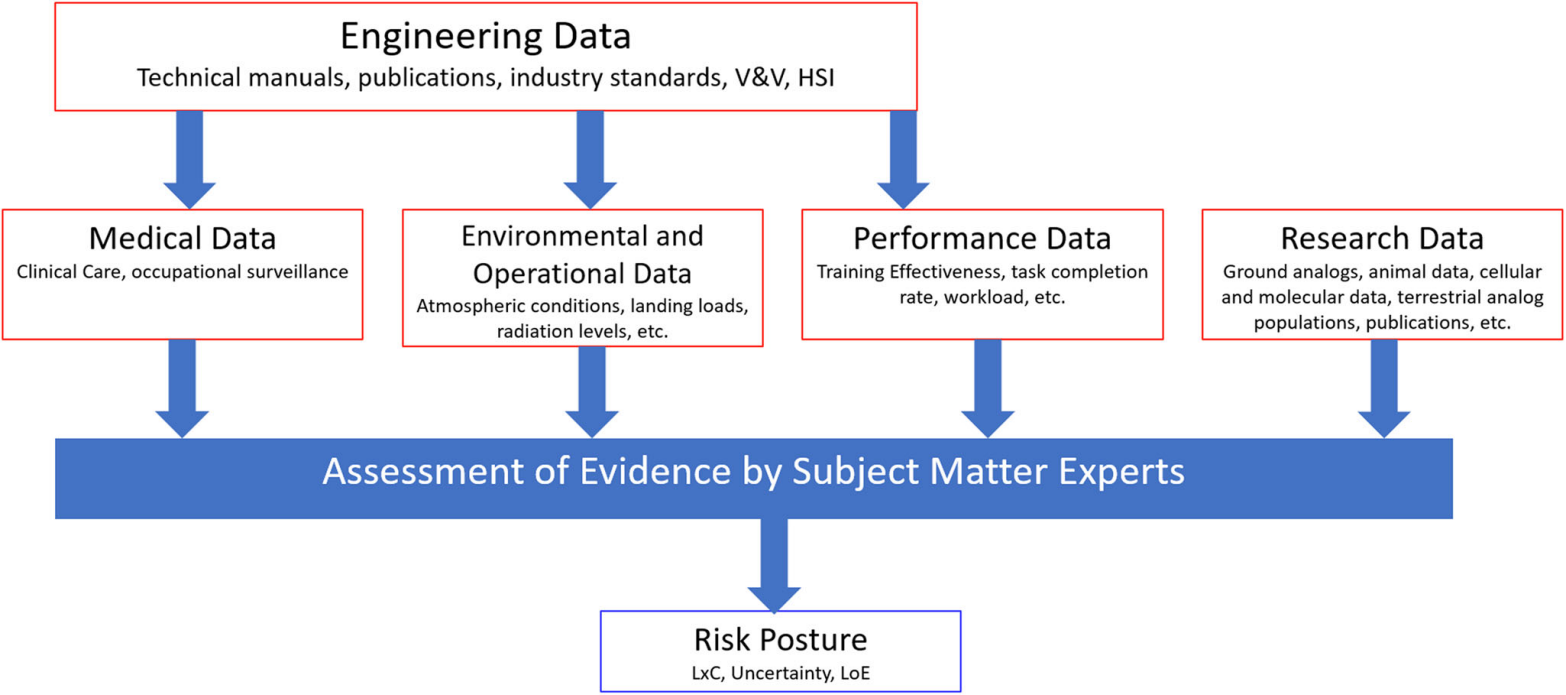
Assessment of evidence. Sources of evidence considered by the HSRB when assigning risk posture. L × C: likelihood vs. consequence. LoE level of evidence^6^.

A team of SMEs called risk custodians, typically comprised of a discipline-specific SME, a physician, and an epidemiologist, evaluate each of the 30 crew health and performance items. This team is responsible for gathering the data that compose the best available set of evidence representing operational, medical, environmental, and occupational surveillance, scientific research, human performance data, and engineering evidence to interpret and support the case for a risk posture (Fig. 1). Over decades of experience in human spaceflight both the methods for data collection and the missions have changed. Different missions have different risk profiles based on spaceflight conditions (number of EVAs, environmental conditions, mission demands, etc.). The Human Research Program (HRP) introduced Standard Measures (https://www.nasa.gov/hrp/i1ymp/spaceflight-standard-measures) in the late 2010s in an attempt to improve data consistency among a broad range of researchers. The role of the epidemiologist on the risk custodian team is to provide the context and metadata where available to the risk custodian team in part so that reasonable assessments of QoE and LoE can be approached.

Risk posture is the HSRB’s understanding, based on the best available evidence, of the likelihood, consequence, and risk disposition of a human system risk for a given mission type^6^. Within NASA, the HRP evidence reports, a review of published evidence related to a particular risk, and the occupational surveillance data in NASA’s Lifetime Surveillance of Astronaut Health database may serve as primary sources for human health and performance evidence based on interpretation by scientists. Relevant terrestrial literature is also considered when setting a risk posture.

### Assessing the Quality of Evidence (QoE)

Assigning QoE is the first part of the evaluation process for studies or data that will contribute to LoE. The QoE helps establish the degree to which specific evidence is applicable to the problem at hand. For example, it is well understood that research in animals has significant limitations when attempts are made to generalize to humans. However, this type of research is often included as part of the risk assessment evidence because it can significantly contribute to the current understanding of biological changes that occur during spaceflight, and the HSRB recognizes that it must work from the evidence available, not the evidence it wishes it had.

For human clinical evidence, the risk custodians are expected to apply the AHRQ grading of evidence approach to assess QoE, as discussed above^25^. To that end, there is a hierarchy of types of studies and the quality of evidence the SMEs and risk custodians consider. Traditionally, meta-analyses are believed to provide a higher quality of evidence than double-blind randomized control trials, which are superior to observational cohorts, which are superior to case studies, etc. Unfortunately, no large meta-analyses or randomized double-blind clinical trials exist for human spaceflight due in part to logistical challenges and ethical issues. Instead, innovative approaches to longitudinal^26,27^ and small-*n* studies^17^ that apply to spaceflight have been developed but are not the traditional targets for AHRQ grading. In these cases, risk custodians are asked to use a binary grading system of “high” and “low” for QoE as an initial step to support the LoE assessment. In the context of QoE, “high” refers to a reliable set of evidence (i.e., Is this animal study worth including in the evidence base because it was a well-designed and executed study?) “Low” then refers to evidence that we may include because there are no better options available. The risk custodian team, tasked by the HSRB to integrate and evaluate the risk evidence, makes this collective SME judgment, which can be deliberated with the HSRB and may be used as a reason for assigning a low LoE score. In practice, not all evidence may be assigned an explicit QoE as it is not recorded in the risk summary template, but it is an integral part of the LoE thought process. The HSRB may use QoE as a reason to prompt inquiry about the appropriate use of specific studies or data to support risk posture or causal assertions.

Although the AHRQ grading system is relevant for clinical studies, it is often difficult to translate to other fields outside of clinical medicine. Animal research has historically been a cornerstone of biological spaceflight research^28–32^. However, an understanding of the pathophysiology of animal models is important to determine their relevance to humans. Given the obvious differences between humans and animals, these studies are classified as “low” by default, although higher levels may be assigned based on the SME review. However, exceptionally high-quality animal or cellular research that adheres to best practices could be considered for a “high” QoE and could then contribute to LoE evidentiary criteria such that assignment above the level of a “weak” LoE is possible. Table 1 shows these best practices.

**Table 1:** LoE and animal studies

	Considerations for increasing the level of evidence recommendations for animal studies
1.	Studies demonstrate relevant assessment and selection of animals (e.g., animal age that translates to relevant to human age translation; performance screening—higher performing animals selected similar to astronauts; maintaining a regimen of exercise and “fitness” levels).
2.	Studies carefully match experiment and control groups (e.g., sex, age, and other characteristics).
3.	The investigators have provided evidence that appropriate animal strains are studied for the question being asked (e.g., for almost all behavioral assessments, researchers continue to use inbred isogenic strains such as C57BL/6 mouse strains, but these can have both genetic and behavioral differences if they come from different breeders and that should be addressed).
4.	Blind coding of all analyses is performed (e.g., evidence coding of data by someone other than the researchers so analysis can be performed in an unbiased manner).
5.	Statistical approaches are rigorously conducted and adequately documented in the “Methods” sections.
6.	Results from independent cohorts collected at different times from different laboratories are explicitly considered.
7.	Multiple outcome measures are used, including measures that are functionally relevant to humans.
8.	Animal models are regularly tested for quality control (e.g., genetic drift, loss of phenotype) and adequately documented in methods sections.
9.	Evidence of validation across models and in the human condition is presented, and literature is referenced to support this.
10.	Any negative data is considered and addressed (e.g., false negatives/false positives), and study limitations are documented.
11	Any evidence or data that seems to contradict the research being represented is documented in the limitations sections.
12	Any failure in translation should be addressed within the context of the following: Was it the animal model itself, the analysis, the clinical trial, or another factor? This should be explicitly considered in the limitations section of published literature.

Studies that demonstrate attention to experimental design and external validation of animal translational research methods contribute to a higher LoE value. Such studies use animal models or cellular or molecular endpoints to generalize from animal to human^6,28–32,41–45^.

The SME evaluates an individual published study and critically appraises the study for bias and limitations, then assigns a QoE score of low or high to denote the relative weight stakeholders should give this individual study. Based on the QoE score and the results of all available evidence, the risk custodian team assigns a LoE score that informs risk stakeholders of the level of certainty behind risk posture assertions.

QoE is used in practice because it is often necessary to explain why a specific paper or data set should *not* be heavily relied upon to influence risk decisions. Limitations sections for papers published in the literature are often overlooked when considering the strength of evidence, and human spaceflight has significant limitations in providing high-quality evidence for risk-informed decision-making. For example, engineers can test their systems to failure while human health researchers cannot, generalizing from animal data to human outcomes is fraught with difficulty, and the “small-n” problem is a chronic challenge in human spaceflight research^17,33,34^.

### Assigning Level of Evidence (LoE)

The LoE scoring system used by the HSRB was derived from a modified set of A. Bradford Hill’s causal guidelines^35^. After the SMEs assess the QoE, the risk custodian team assigns a numerical score for LoE for the overall risk and a risk posture for their specific risk as applied to each DRM. For a spaceflight-induced risk to humans, the fundamental question is causal, i.e., what aspects of the spaceflight environment cause changes in the human system that elevate the risk of the undesired Mission Level Outcomes? An understanding of these effects drives an understanding of mission-level outcomes. The Hill guidelines list 9 aspects of causal relationships against which evidence might be weighed to determine causation. The HSRB includes 6 of these aspects: temporality, analogy, mechanism, reproducibility, specificity, and coherence, each of which is defined in Table 2. The HSRB does not include the other 3, which are the traditional terrestrial criteria—strength, biological gradient, and experiment—because while they support causal conclusions, they are neither necessary nor sufficient to establish it and are therefore viewed as non-essential^35^. The causal relationships embodied in the evaluated set of evidence are captured in each risk DAG diagram within the network of risk-contributing factors that lead to the outcomes of interest.

**Table 2:** Causal guidelines

Criterion	Definition	Notes
Temporality	Causes must precede effects, including any delay that may be expected between them.	This is necessary for all posited causal effects, even speculative ones.
Analogy	A proposed causal relationship should have some similarity to a known process or circumstance.	Analogs can make substitutions in one or more organisms, settings, specific exposures, or outcomes.
Mechanism	A modification of Hill’s original “Plausibility.” Causal relationships should have a plausible theoretical explanation.	This can be in terms of physiology, cellular processes, ‘omics, and more.
Reproducibility	The attribution of causation is strengthened when results can be replicated by different investigators across different times and places with different study subjects.	
Specificity	Causal explanations are stronger when the causal relationship is observed in specific contexts, with specific persons, exposures, and outcomes.	This is the classic Person/Place/Time of epidemiology.
Coherence	The agreement between all evidence, especially when it validates proposed mechanisms.	This is translational science.

Causal guidelines employed by the HSRB for the level of evidence assessment (adapted from Sir A. Bradford Hill)^6,35^.

To assign a LoE, a causal relationship must be established. Using the Hill-inspired guidelines, the LoE is evaluated and scored as either speculative (4), weak (3), moderate (2), or strong (1). The progressive requirements are shown in Table 3.

**Table 3:** LoE assignment guidelines

LoE	Temporality	Analogy	Mechanism	Reproducibility	Specificity	Coherence
4—Speculative	X	X				
3—Weak	X	X	X for 1 or 2 of these			
2—Moderate	X	X	X	X	X	
1—Strong	X	X	X	X	X	X

Numerical assignments for LoE are determined based on the Hill guidelines. An LoE is assigned as speculative if it meets both the temporality and analogy criterion. The addition of any one or two of the mechanisms, reproducibility, or specificity criteria justifies an assignment of weak LoE, whereas all 3 criteria are necessary for a moderate LoE. A strong LoE must meet all six criteria^6^.

Level 4, the lowest LoE score, is titled “speculative”. Speculative denotes possible causal relationships that make theoretical sense but have little to no evidence to support the assertion. This may be because the evidence is primarily from terrestrial analogs of spaceflight that may not adequately reflect environmental exposures in space. To be included as a relationship on the DAG at the speculative level, the evidence must meet the definition of temporality and analogy: Temporality is where the cause must precede the effect; and Analogy is when similar situations or existing terrestrial knowledge helps inform a hypothesis for potential mechanism. Information for this category may include spaceflight analogs but can also include occupational cohorts, and laboratory or animal studies that are unrelated to spaceflight. Both elements are required for the speculative level because without the proper temporal sequence, no causation is possible, and without at least analogy, accepting the temporal sequence as causal may simply be a *post hoc* fallacy.

Level 3, the second-lowest score, is titled “weak”. Causal effects that are not well-understood epidemiologically or mechanistically are typically assigned this level. To meet this criterion, the evidence must meet the criteria for a speculative LoE (temporality and analogy), as well as have at least one, but not all, of the following: mechanism, reproducibility, and specificity. Mechanism denotes a probable but not necessarily proven biological chain of events. Evidence may exist only in animal studies or at the cellular level. All animal studies showing a biological mechanism are automatically rated weak until further evaluation can support higher levels. Next, Reproducibility improves the LoE because the evidence is always improved if repeated in other investigations. Specificity denotes evidence that has been focused on a particular person, place, or time matching that of the astronaut cohort. A weak LoE score indicates a possible cause and effect between the hazard(s) and the claimed effects on humans that may lead to clinically and operationally meaningful levels of concern for the HSRB. If the QoE assessment of relevant publications is weak, the LoE should remain either weak or speculative. Weak evidence requires any one, or a combination, of mechanism, reproducibility, or specificity to be met. If all 3 are supported, then the LoE should be scored as moderate (2). For communication in high-level discussions, levels 3 and 4 may be occasionally grouped together under the single title “weak”.

Level 2, the second highest LoE, is titled “moderate”. A moderate LoE must meet all the above-mentioned criteria: temporality, analogy, mechanism, reproducibility, and specificity. The causal effects must have epidemiological evidence, although their biological mechanisms may not yet be fully validated. For animal or cellular evidence to meet the specificity guideline, the studies must demonstrate attention to experimental design and external validation of animal translational research methods, as discussed in Table 1. A moderate LoE indicates a likely cause-and-effect relationship between the hazard(s) and claimed effects on humans. This suggests clinically and operationally meaningful levels of concern and may trigger the creation of a new risk, changes in design requirements, or recommendations for increased risk mitigation efforts.

Level 1. the highest LoE, is titled “strong”. Causal effects that meet these criteria have attained broad-level consensus among SMEs. High-level epidemiological evidence with well-understood mechanisms must be available for humans. All the modified causal criteria, including coherence, are required for this category. Coherence is defined as agreement between laboratory and human-subject results. The QoE for this evidence must be characterized as high to be considered coherent. Furthermore, a strong LoE indicates a deep understanding of the relationship between cause and effect. For risk posture, the relationship between the hazard(s) of spaceflight and implied effects on humans is well understood. For DAGs, there is a strong understanding of the causal relationship between two nodes on a graph.

### Applications of LoE score

The first application of LoE is to assess risk posture for a human system risk, which is used in high-level communication with programs and the agency (the second area is LoE within DAGs and is discussed in the next section). The HSRB tracks and manages 29 risks and their accompanying risk posture^6^ that is based on the LxC (likelihood vs consequence) score and the risk disposition. The L × C score is based on a 5×5 quantitative and qualitative scoring system applied to a risk’s most probable consequence within applicable risk impact categories (in-mission, long-term health, flight recertification) and the associated likelihood assessed against 4 design reference mission (DRM) categories of short and long mission types. The L × C score is supported by an assessment of evidence and plotted in the 5×5 risk matrix that has an associated risk color (red, yellow, green). An accompanying risk disposition represents the board’s recommended agency’s overall position on the state of the risk and further needs as assessed. Table 4 shows the factors relevant to risk assessment, including the DRM, mission type, and duration, L × C score and risk disposition, L × C drivers and assumptions, and risk disposition rationale, which provide brief justification for the L × C score and agency level of acceptance^6^.

**Table 4:** Risk posture summary example

Summary table showing an example risk posture summary for low earth orbit design reference missions (DRM) from the electric shock risk. The area bolded in orange is the likelihood vs. consequence (L × C) and risk disposition for the short-duration DRM. The area bolded shows the level of evidence (LoE) that indicates the level of confidence in the risk score and disposition. Ops: in-mission operations. LTH: long-term health^6^.

Most risks are assessed as impacting two categories of outcomes: in-mission operations (Ops) and long-term health (LTH) of astronauts. These 2 areas of potential risk often have separate evidence, given that Ops is specifically limited to the timeframe between the launch of a mission and a successful return to Earth. Ops examples include risks to hearing from the vehicle noise environment, risks of performance issues related to muscle strength changes from exposure to the altered gravity environment, and risks of injury from Extravehicular Activity operations. The LTH category captures risks associated with the post-flight and post-career health issues that may be encountered long after exposure to the spaceflight environment. LTH examples include the risk of cancer from exposure to the space radiation environment and the risk of bone fractures late in life from the bone changes that occur during exposure to the altered gravity environment. “Risk Posture Level of Evidence” is displayed in 4 places on the risk chart to clearly show the LoE score for a specific risk.

When assessing the overall risk posture for a specific risk, the SMEs must consider 2 questions.

1. What is the LoE of the body of applicable evidence that helps us understand the chances that there will be a consequential problem? (e.g., *Does the evidence suggest there will be a consequential problem?*)
2. What is the LoE of the applicable evidence that helps us understand the chances that with known or anticipated solutions we will be able to successfully mitigate that problem? (e.g., *Does the evidence suggest we will be able to effectively mitigate that problem?*)

This is a sequential process where the LoE score denotes the certainty associated with the answers to these above questions. If the answer to question 1 is that the LoE is “strong” and that a consequential problem is extremely unlikely, this means there is little to no doubt that the likelihood of a consequential problem is extremely low. In this case, the risk posture is assigned green (low risk), and the risk posture is assigned green (low risk). If the LoE is “weak” and uncertainties exist regarding the potential of a problem developing, then this identifies an area worth considering for further characterization investment by NASA, and the risk posture is assigned as either yellow or red (moderate or high-risk). Characterization investment is an investment in research that will help improve the evidence base to determine the potential magnitude of a problem that may be experienced in human spaceflight^6^.

If “strong” evidence exists that a consequential problem will occur, then question 2 becomes relevant. LoE that applies to risk mitigation includes not only clinical evidence that a treatment will work in the spaceflight environment but also the engineering and programmatic evidence that the necessary capability (resources and skills) will be available during a mission to implement the treatment. In the resource-limited environment of spaceflight, it is not possible to carry all the resources that are desired for all the problems that may be encountered. Therefore, clinical evidence of efficacy must be tempered by a new definition of effectiveness that includes programmatic and engineering decisions that may be made years before a flight occurs.

Risk custodian teams return to the HSRB to present risk updates on a periodic basis (annually for red risks, biennially for yellow risks, and as needed for timely/event-driven updates) and can present new evidence supporting their assigned risk at those times. The opportunity for ‘As Needed’ updates is at the discretion of the Risk Custodian team when they recognize important new evidence is available that should be considered by the board. When new evidence is brought forward, the SMEs should provide a summary of the evidence, its quality, and insight into any effect on LoE. Changes proceed through a configuration management process to formally track the progress of each risk over time^6^.

The second application of LoE is assigning metadata to the edges in DAGs. DAGs are more completely discussed in DAG Documentation Guidance^20^. These are described here briefly to illustrate the application of LoE. DAGs are, first and foremost, intended as a communication tool to help SMEs from disparate fields establish and work from a shared mental model of how and where risk arises during human spaceflight. Causal DAGs are network diagrams that show unidirectional representations of causal flow^36–38^. (While feedback loops are important parts of realistic descriptions of events, they can be represented by time-indexing that retains the acyclic structure used here.) The problem of interest for human spaceflight is related to the effects of the spaceflight environment on humans and the limitations in mitigating risk due to engineering constraints imposed by the vehicle, suit, or habitat design and operation. A unidirectional flow of risk is assumed to begin when an astronaut is immersed in the spaceflight environment. Each unidirectional arrow (edge) connects one variable (node) to another, indicating that the probability distribution of the second node is dependent on the value of the first. The initial nodes for each of the human system risks are always a subset of the five human spaceflight hazards that pose a danger to human health. From the time of launch, exposure to environmental hazards begins a series of physiologic changes to the human astronauts. Distance from Earth is a unique hazard that influences the mass, power, and volume allocations of the vehicle, which then affects the systems and countermeasures that are available for risk reduction during a mission. The 5 hazards account for both the human changes that must be faced as well as the engineering constraints on the mass, power, and volume allocations of the vehicle. The causal flow of risk for each DAG terminates at the five mission level outcomes: task performance, loss of mission objective, loss of mission, loss of crewmember life, and LTH outcomes. Mission-level outcomes represent specific agency-level consequences that have an associated likelihood described by the L × C matrices used by the HSRB. In between the initial hazards and the outcomes are important factors known or hypothesized to propagate or mitigate risk through the system. These include contributing factors, countermeasures intended to reduce risk, and the influence of other human system risks. Essentially, these diagrams are a picture created by the HSRB community that represents the flow of risk as currently understood. Each edge connecting 2 nodes is a falsifiable hypothesis that can be supported or refuted by evidence. DAGs are intended to be dynamic visualizations such that as new evidence is brought forth, the diagrams can be updated to reflect the current understanding of the causal flow of risk. This relationship with evidence can be further visualized by using a numeric LoE assessment to weigh the edges of the DAG and differentiate areas of strong evidence from those with weaker evidence.

**Fig. 2:**
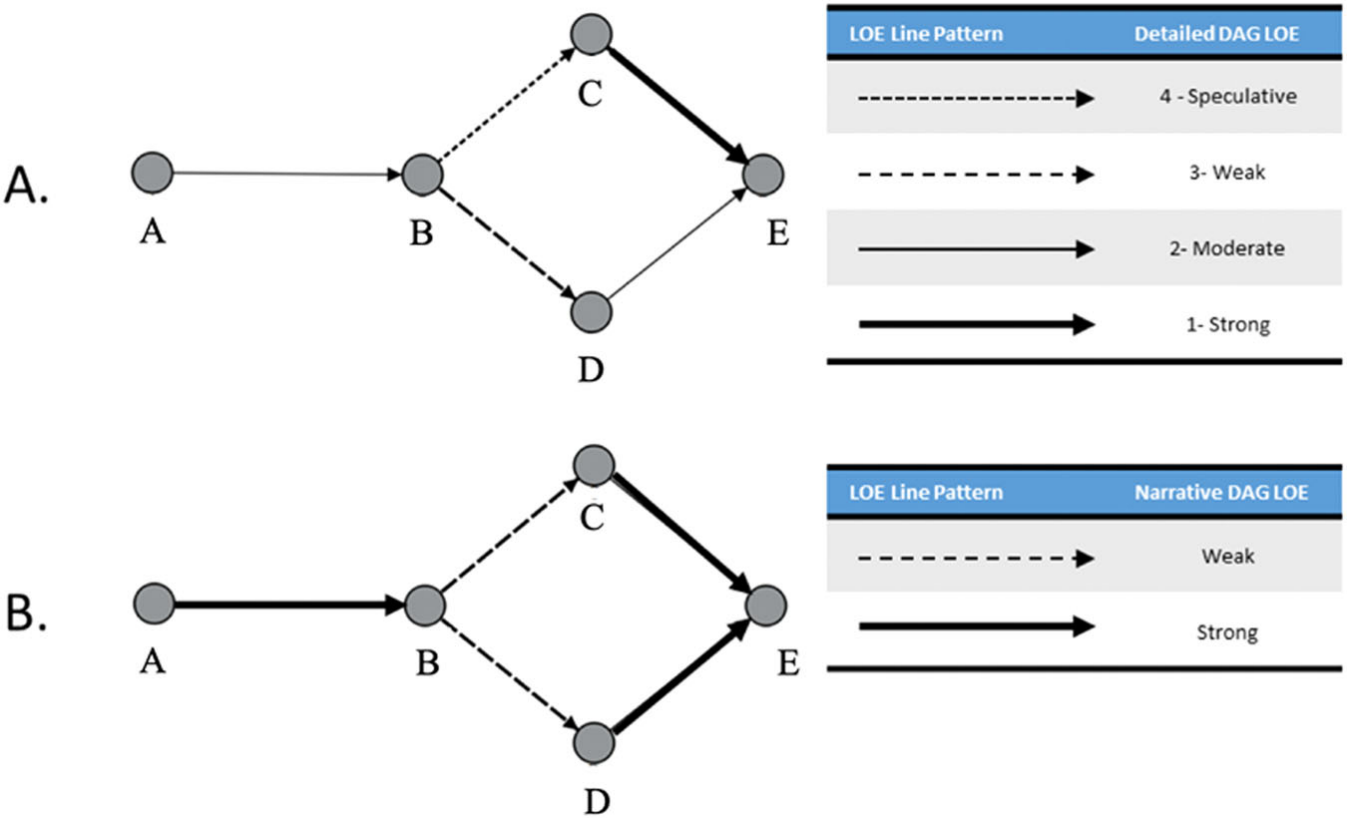
Simplified and detailed DAG LoE examples. Example of a DAG showing LoE scores via different thicknesses and styles of arrows. **A** Shows an example of a detailed DAG LoE assignment that illustrates all 4 levels. **B** Shows a simplified version, used to help facilitate high-level discussions, that merges levels 3 and 4 into “weak” and levels 1 and 2 into “strong”^6^.

The HSRB uses 2 versions of DAGs: narrative and detailed. Narrative DAGs are used as high-level communication tools, whereas detailed DAGs are intended to show a level of detail that is appropriate for deeper discussion by SMEs. In the narrative DAGs, steps in the biological or engineering process may be condensed into a single category node to minimize visual complexity. To keep the high-level narrative DAGs as simple as possible, only 2 LoE scores are used: “strong” (includes strong and moderate LoE) and “weak” (includes weak and speculative LoE). Figure 2 illustrates the difference between LoE on the 4-level scale for detailed information (panel A) and the strong vs. weak scale for high-level communication needs (panel B). The simplified narrative format is used for communication with program directors and executives to minimize the distractions of excessive detail. The detailed DAGs are used by the risk board when discrete intermediate steps between hazards and outcomes must be visualized for a complete understanding of the risk. The detailed DAG also uses the 4-level LoE scale to demonstrate potential gaps in knowledge or capability and to provide additional granularity in visual communication.

In some cases, a causal link in a DAG is considered strong “by definition”. There are several categories of causal assertions that are understood to be strong LoE “by definition”. For example, all DAGs include nodes for vehicle design, crew health and performance systems, and certain medical capabilities. It is well understood that decisions made during the vehicle design process will determine the mass and volume allocations for all systems and, by extension, affect the probability that specific countermeasures and capabilities will be available to the crew during a mission. This means, from an evidence perspective, that the LoE of certain edges strongly affects the path to downstream nodes. These can be thought of as “by design”. Additionally, the well-understood biological factors unrelated to vehicle/system design are assigned as “by definition”. For example, the individual factors node, which includes factors such as age, sex, height, and genetic predisposition, is known to contribute to individual biological responses to the spaceflight environment. In the case of the loss of mission objectives node, a particular number of lost mission objectives will inevitably lead to the outcome loss of mission “by definition”. Assigning “by definition” to edges within the DAGs in a consistent manner removes the burden of each risk team having to prove the obvious and focuses the SMEs on more uncertain areas by reducing the number of edges to be researched, as well as providing consistency between the DAGs. “By definition,” connections are automatically scored as strong.

**Fig. 3:**
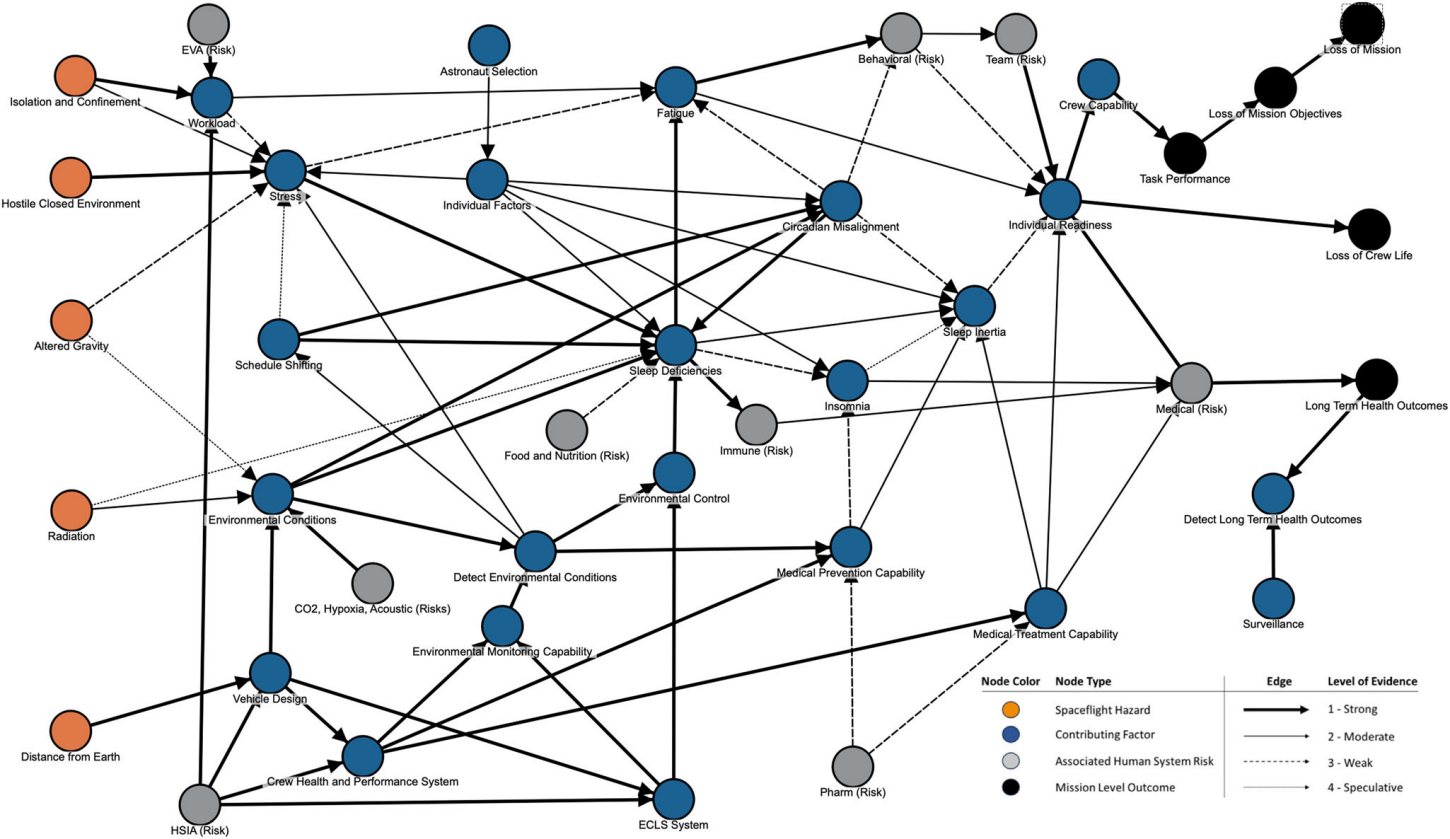
Sleep risk DAG example. Sleep risk DAG (last updated May 13, 2022) showing the level of evidence assignments (strong, moderate, weak, and speculative). EVA extravehicular activity, CO_2_ carbon dioxide, HSIA human system integration architecture, ECLS environmental control and life support, Pharm pharmaceutical.

Figure 3 is an example of an HSRB DAG that illustrates the application of LoE to a human system risk. This DAG describes the *Risk of Performance Decrements and Adverse Health Outcomes Resulting from Sleep Loss, Circadian Desynchronization, and Work Overload* (Sleep Risk).

The left side of the DAG starts with the spaceflight hazards shown in orange, and it terminates on the right side at mission-level outcomes, which are shown in black. Blue nodes show contributing factors such as environmental conditions, physiologic or anatomic changes, or medical treatment capabilities. If any of the other 28 human spaceflight risks are relevant, they are shown as gray nodes, which have entirely separate DAGs. In practical application, one or more pertinent nodes from the associated DAG interface with the risk shown in the current DAG.

To illustrate the utility of the DAGs and the LoE assessment at the detailed level, consider the “strong” links between the environmental conditions node and the sleep deficiencies node. For some missions, environmental controls may be included in the vehicle design, the environmental control and life support (ECLS) system, and the crew health and performance system. These links are all shown as “strong” LoE because, *by definition*, many aspects of designs are known to affect the crew’s likelihood and severity of sleep deficiencies.

**Fig. 4:**
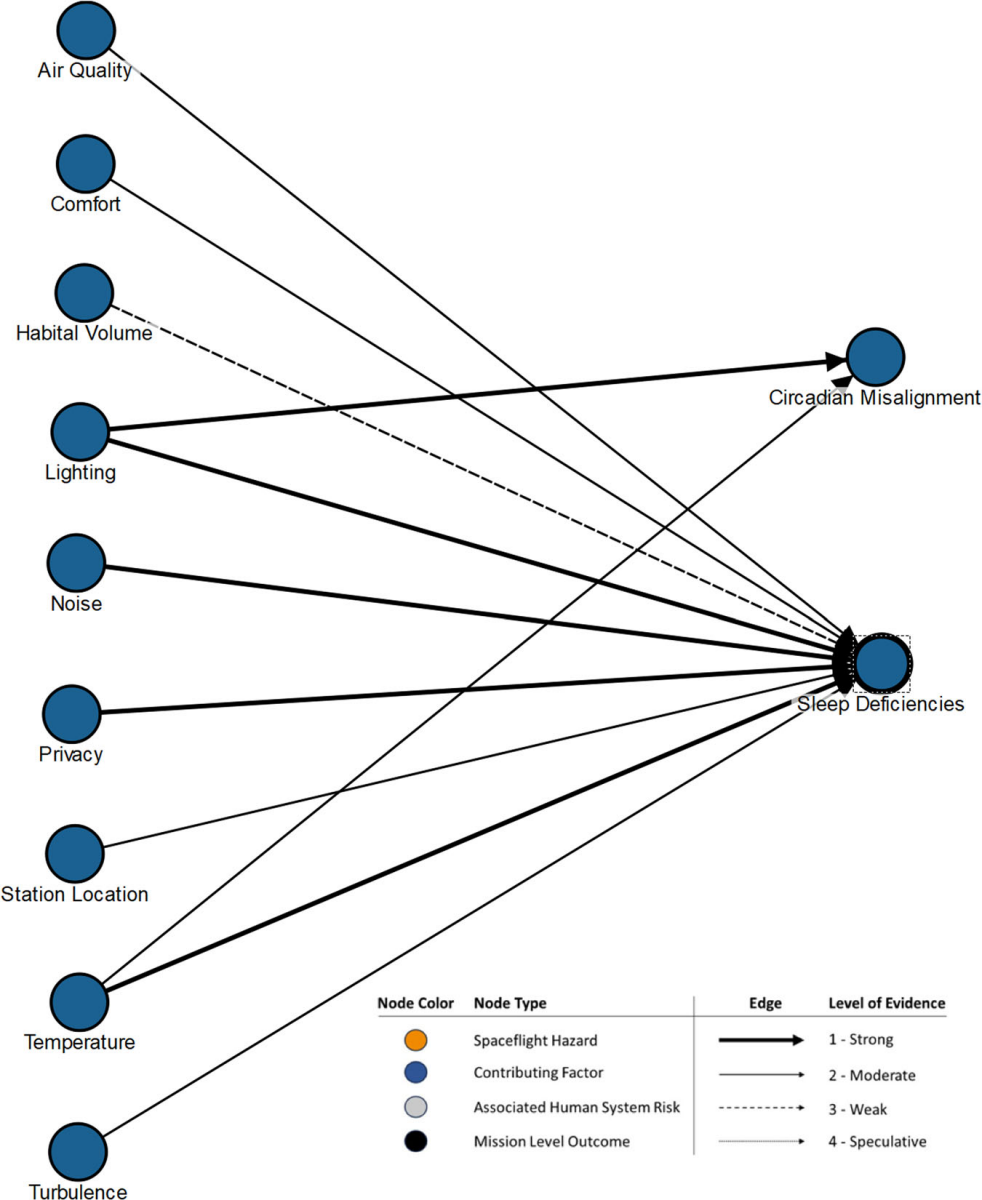
Sub-DAG example. Sub-DAG from the Sleep DAG showing an expansion of the environmental conditions node into relevant nested nodes with level of evidence (LoE) assignments that visually display how those contributing environmental factors causally relate to sleep deficiencies and circadian misalignment. Detailed LoEs are displayed.

Expanding the environmental conditions node to a deeper level reveals multiple nodes nested under that category mode, as shown in Fig. 4. These nested nodes are taken from evidence assessments of the factors that can be manipulated to improve the sleep environment for astronauts^39,40^. Although it is beyond the scope of this paper to describe the relationships within the DAG in great depth, this example uses LoE assignments to show which of those factor’s causal influences on sleep deficiencies and circadian misalignment are well or poorly understood. At the detailed level the LoE assignment provides visual insight into the gaps in knowledge that exist. The uncertainty conveyed by these edges highlights areas where characterization efforts, technology development, or design requirements may be needed to reduce risk in future missions.

## Outlook and summary

Risk posture is the assessment of all the available knowledge about a specific spaceflight risk and represents the highest level of communication used within NASA to inform stakeholders of the risks as they are currently understood. Risk posture is based on the LxC Matrix, which determines the level of risk the agency acknowledges. LoE in the context of risk posture denotes uncertainty about that assessment. An assessment of LoE is required for each risk update and for each DRM and is configuration managed at the HSRB.

NASA uses LoE in two different applications that have different purposes and meanings, as reviewed here—the risk posture represented by the likelihood by consequence score (L × C) and the DAGs. For the purposes of risk posture, the LoE scores indicate the uncertainty in the relationship between the evidence base and the L × C assigned by the HSRB for a given DRM. For the DAGs, the LoE score describes how well we understand the causal relationship between two nodes and asserts the level of confidence that the causal link truly exists.

Although the HSRB does not require LoE assignments to risk DAGs and they are not configuration managed, DAGs are currently derived and used to seek deeper insight into the knowledge base. Performing a full LoE assessment for a DAG has several advantages. First, it forces SMEs and risk custodian teams to identify how well they think they understand the causal links between the factors that contribute to the risk. Second, it identifies gaps in knowledge and characterizes the certainty of assumptions in evidence. This ensures that assumptions are recorded, assessed critically, and brought to the awareness of the HSRB.

The strengths of this method of LoE assessment are in the insights and conversations it generates. Appropriate interpretation of evidence allows everybody, regardless of their level of knowledge in the given area, to discuss QoE and LoE using the same mental model. These conversations can uncover areas in a DAG where evidence is lacking, allowing a more standardized assessment of whether a specific gap in knowledge should be prioritized for research investments. A high LoE (implying low epistemic uncertainty) indicates confidence in the underlying recommendations conveyed for spacecraft design, operations, and trade-space decisions.

The limitations of this approach include perceived subjectivity. Individual subjectivity is addressed through the conversation and agreements generated within the HSRB structure. While this does not negate the possibility of error, the continuous risk management process enables opportunities to continuously challenge or affirm existing conclusions. Over time, this should systematically reduce the error that influences risk-informed decision-making. In this sense, the continuous risk management process is a Bayesian learning process that helps check a prior belief or error. Another limitation is the availability of relevant data and evidence, given the small number of people who have flown in space. This limitation is real and is also the primary motivation to use a Bayesian learning process as the frequency of human spaceflight increases.

For the DAGs, it is important to understand that LoE coveys the level of understanding of the causal link in question. For example, how much evidence is there that radiation affects sleep? In this sense, a speculative or weak LoE identifies a gap in knowledge. Gaps in knowledge are particularly useful to identify as they may help inform research prioritization.

The challenges that will be faced as space missions move further from Earth for longer durations are unprecedented in the human experience. NASA recognizes that a limited human health and performance evidence base is available to support recommendations for planners and directors of these missions. Despite this, the pace of human spaceflight missions is increasing, and the need is increasing for agency-driven risk-informed recommendations on countermeasures that ensure safe exploration missions^7^. The systems engineering decisions that are made today based on current knowledge will have far-reaching consequences for human spaceflight over the next decade.

In summary, despite the immense complexity of space vehicles and the humans who go to space, the systems for communicating risk must be simple and transparent. An assessment of LoE is critical to the discussion of uncertainty surrounding risk. The LoE system discussed here is a rudimentary way to communicate our level of understanding of risk posture and causal inference so that decision-makers such as mission planners and program directors receive adequate insight into risks and are not overwhelmed with excessive, potentially uninformative information. The goal of this LoE process is to provide a systematic and repeatable approach to identify and improve risk postures for the 29 human system risks that are managed by the HSRB. Similarly, the application of LoE scores to the risk DAGs is intended to visually communicate the weak areas in understanding and evidence and to aid in planning and investment decisions to close those gaps. In the context of the larger set of risk management tools and processes managed by the HSRB, this can be a useful tool for program development. The visual identification of the speculative and weak LoEs within the DAGs can methodically improve understanding of the interactions among the numerous factors contributing to the risk to human health and performance and help develop effective mitigation strategies.
